# Genome Sequence of *Photobacterium halotolerans* MELD1, with Mercury Reductase (*merA*), Isolated from *Phragmites australis*

**DOI:** 10.1128/genomeA.00530-15

**Published:** 2015-06-04

**Authors:** Dony Chacko Mathew, Gincy Marina Mathew, Ronnie Gicaraya Gicana, Chieh-Chen Huang

**Affiliations:** a Department of Life Sciences, National Chung Hsing University, Taichung, Taiwan; b School of Biosciences, Mar Athanasios College for Advanced Studies (MACFAST) Biocampus, Tiruvalla, Kerala, India; c Department of Plant Pathology, National Chung Hsing University, Taichung, Taiwan

## Abstract

Here, we present the whole-genome sequence of *Photobacterium halotolerans* strain, MELD1, isolated from the roots of a terrestrial plant *Phragmites australis* grown in soil heavily contaminated with mercury and dioxin. The genome provides further insight into the adaptation of bacteria to the toxic environment from where it was isolated.

## GENOME ANNOUNCEMENT

*Photobacterium* spp. are Gram-negative bacteria belonging to the family *Vibrionaceae*. Though they are found to be primarily associated with marine environments (1), *Photobacterium halotolerans* MELD1 was isolated from the rhizosphere of a terrestrial weed, *Phragmites australis*, found growing in mercury- and dioxin-contaminated land located near the seacoast (2). In our previous study, we demonstrated that *P. halotolerans* MELD1 helped with the phytoprotection of *Vigna unguiculata* from mercury stress (2). To gain insight into the genetic traits among the closely related *P. halotolerans* strains, whole-genome sequencing of terrestrial environment dwelling *P. halotolerans* MELD1 was performed.

The MELD1 whole-genome sequence was obtained using Illumina technology. Ten micrograms of total DNA was sonicated by a Misonix 3000 sonicator to sizes ranging from 400 to 500 bp. DNA sizing was checked by a bioanalyzer DNA 1000 chip (Agilent Technologies, Santa Clara). One-microgram sonicated DNA was end repaired, A tailed, and adaptor ligated following Illumina’s Trueseq DNA preparation protocol. The sequences generated went through a filtering process to obtain the qualified reads. ConDeTri (3) was implemented to trim or remove the reads according to the quality score. Cleaned and filtered nuclear reads were assembled *de novo* using ABySS (4). Genome annotations were created in MAKER 2.00 (5) using a GeneMark (6) model trained for MELD1 via self-training. The resulting predictions were searched against the NCBI nonredundant (nr) database by using BLASTp.

The whole-genome draft of *P. halotolerans* MELD1 consists of 57 contigs for a total of 4,758,037 bp with an overall G+C content of 51%, 258 pseudogenes, 17 rRNA genes, and 88 tRNA genes. In our previous work, we identified the presence of a *merA* gene and its mercury reductase activity, as well as resistance to toxic compounds like cadmium, lead, and dioxin (2). As a trait of adaptation to the mercury-contaminated habitat, the genome of MELD1 contains a *mer* operon containing a mercury reductase gene (*merA*). Furthermore, MELD1 had a cluster of genes responsible for stress resistance, multidrug efflux pumps, and aerobactin siderophore. They also bear *lux* genes and genes responsible for gamma aminobutyric acid (GABA) (7) and pyrroloquinoline quinone (PQQ) (8). These unique characteristics make the strain *P. halotolerans* MELD1 an effective plant growth-promoting bacterium in heavy metal-contaminated environments.

### Nucleotide sequence accession numbers. 

The whole-genome sequence of *P. halotolerans* MELD1 was deposited at DDBJ/EMBL/GenBank under the accession no. JWYV00000000. The version described in this paper is the first version, JWYV01000000.

## References

[1] Deep K, Poddar A, Das SK 2014 *Photobacterium panuliri* sp. nov., an alkalitolerant marine bacterium isolated from eggs of spiny lobster, *Panulirus penicillatus* from Andaman Sea. Curr Microbiol 69:660–668. doi:10.1007/s00284-014-0638-0. 24962598

[2] Mathew DC, Ho Y-N, Gicana RG, Mathew GM, Chien M-C, Huang C-C 2015 A rhizosphere-associated symbiont, *Photobacterium* spp. strain MELD1, and its targeted synergistic activity for phytoprotection against mercury. PLoS One 10:e0121178. doi:10.1371/journal.pone.0121178. 25816328 PMC4376707

[3] Smeds L, Künstner A 2011 ConDeTri—a content dependent read trimmer for Illumina data. PLoS One 6:0026314. doi:10.1371/journal.pone.0026314. PMC3198461 22039460

[4] Simpson JT, Wong K, Jackman SD, Schein JE, Jones SJM, Birol I 2009 ABySS: a parallel assembler for short read sequence data. Genome Res 19:1117–1123. doi:10.1101/gr.089532.108. 19251739 PMC2694472

[5] Holt C, Yandell M 2011 MAKER2: an annotation pipeline and genome-database management tool for second-generation genome projects. BMC Bioinformatics 12:491. doi:10.1186/1471-2105-12-491. 22192575 PMC3280279

[6] Lukashin AV, Borodovsky M 1998 GeneMark.Hmm: new solutions for gene finding. Nucleic Acids Res 26:1107–1115. doi:10.1093/nar/26.4.1107. 9461475 PMC147337

[7] Misra HS, Rajpurohit YS, Khairnar NP 2012 Pyrrolquinoline-quinone and its versatile roles in biological processes. J Biosci 37:313–325. doi:10.1007/s12038-012-9195-5. 22581337

[8] Dagorn A, Chapalain A, Mijouin L, Hillion M, Duclairoir-Poc C, Chevalier S, Taupin L, Orange N, Feuilloley MGJ 2013 Effect of GABA, a bacterial metabolite, on *Pseudomonas fluorescens* surface properties and cytotoxicity. Int J Mol Sci 14:12186–12204. doi:10.3390/ijms140612186. 23743829 PMC3709781

